# A Critical Comparison Among High-Resolution Methods for Spatially Resolved Nano-Scale Residual Stress Analysis in Nanostructured Coatings

**DOI:** 10.3390/ijms26073296

**Published:** 2025-04-02

**Authors:** Saqib Rashid, Edoardo Rossi, Spyros Diplas, Patricia Almeida Carvalho, Damian Pucicki, Rafal Kuna, Marco Sebastiani

**Affiliations:** 1 Department of Civil, Computer Science and Aeronautical Technologies Engineering, Roma TRE University, Via Della Vasca Navale, 62, 00146 Rome, Italy; edoardo.rossi@uniroma3.it (E.R.); marco.sebastiani@uniroma3.it (M.S.); 2 SINTEF Industry, Forskningsveien 1, 0373 Oslo, Norway; spyros.diplas@sintef.no (S.D.); patricia.carvalho@sintef.no (P.A.C.); 3 Department of Nanometrology, Faculty of Electronics, Photonics and Microsystems, University of Science and Technology, 50-372 Wroclaw, Poland; damian.pucicki@port.lukasiewicz.gov.pl; 4 Łukasiewicz Research Network—PORT Polish Center for Technology Development, Stabłowicka 147, 54-066 Wrocław, Poland; rafal.kuna@port.lukasiewicz.gov.pl

**Keywords:** AlGaN/GaN multilayer coatings, MOVPE deposition, HRSTEM-GPA, FIB-DIC residual stress measurement, high spatial resolution techniques

## Abstract

Residual stresses in multilayer thin coatings represent a complex multiscale phenomenon arising from the intricate interplay of multiple factors, including the number and thickness of layers, material properties of the layers and substrate, coefficient of thermal expansion (CTE) mismatch, deposition technique and growth mechanism, as well as process parameters and environmental conditions. A multiscale approach to residual stress measurement is essential for a comprehensive understanding of stress distribution in such systems. To investigate this, two AlGaN/GaN multilayer coatings with distinct layer architectures were deposited on sapphire substrates using metalorganic vapor phase epitaxy (MOVPE). High-resolution X-ray diffraction (HRXRD) was employed to confirm their epitaxial growth and structural characteristics. Focused ion beam (FIB) cross-sectioning and transmission electron microscopy (TEM) lamella preparation were performed to analyze the coating structure and determine layer thickness. Residual stresses within the multilayer coatings were evaluated using two complementary techniques: High-Resolution Scanning Transmission Electron Microscopy—Graphical Phase Analysis (HRSTEM-GPA) and Focused Ion Beam—Digital Image Correlation (FIB-DIC). HRSTEM-GPA enables atomic-resolution strain mapping, making it particularly suited for investigating interface-related stresses, while FIB-DIC facilitates microscale stress evaluation. The residual strain values obtained using the FIB-DIC and HRSTEM-GPA methods were −3.2 × 10⁻^3^ and −4.55 × 10⁻^3^, respectively. This study confirms that residual stress measurements at different spatial resolutions are both reliable and comparable at the required coating depths and locations, provided that a critical assessment of the characteristic scale of each method is performed.

## 1. Introduction

The residual stresses in multilayer thin coatings are strongly dependent on several factors such as layer thickness and numbers, coating and substrate materials, thermal expansion coefficient mismatch between substrate and coating material, deposition techniques, process parameters, and working pressure [1,2,3,4]. These stresses are critical in multilayer structures and play an essential role in the performance and reliability of coatings, impacting mechanical and physical properties like hardness, adhesion, fracture, fatigue, and corrosion resistance. Excessive residual stresses can lead to coating delamination, cracking, or deformation, thereby compromising the structural integrity and functional properties of the coating system [5]. In contrast, controlled residual stresses can enhance adhesion, improve wear resistance, and optimize mechanical performance, particularly in applications such as protective coatings, microelectronics, and optical films [6]. Therefore, a thorough understanding and precise control of residual stress distribution at multiple scales are essential for ensuring the reliability and longevity of multilayer coatings in advanced engineering applications. To achieve this, it is essential to understand the origin and evaluation of residual stress in coatings by means of suitable and reliable techniques and to develop methods to predict and control them to be able to design multilayer films with high performance [7]. The residual stresses are determined in the form of elastic strain, and for analyzing the depth profile of residual strain, it is crucial to understand the in-depth lattice strain functions [8,9].

It is very crucial to accurately quantify residual stresses in nanoscale multilayer coating systems, bridging the gap between local lattice distortions and microscopic stress accumulation. Currently, residual stress measurements are typically conducted using more advanced techniques that offer spatial resolution ranging from micron to sub-nano level. These techniques are generally classified into three categories: non-destructive, destructive, and semi-destructive techniques. Non-destructive residual stress measurement includes the ultrasonic technique; which utilizes the propagation of high-frequency sound waves through materials where wave velocity corresponds to the residual stresses within the coatings [10,11,12], laser acoustic wave technique; uses the interaction of laser-generated ultrasonic waves with the material to assess the residual stress state in thin films and coatings [13,14], x-ray diffraction technique; is a powerful tool for measuring residual stresses in multilayer thin films, providing a non-destructive and accurate way to analyze stresses both at the surface and within the material [15,16,17,18], the Raman spectroscopy technique; is a highly effective technique for measuring residual stresses in thin films and coatings due to its high spatial resolution, non-destructive nature, and versatility across different materials [19,20,21,22], and high-resolution scanning transmission electron microscopy—graphical phase analysis (HRSTEM-GPA); a specialized technique that integrates the high spatial resolution of STEM with the phase analysis capabilities of GPA to analyze strain and stress distributions within thin films and coatings at the nanoscale [23,24,25,26,27]. Destructive techniques consist of the counter method; which involves measuring the deformation after material removal that reflects the residual stress, the crack compliance method; based on measuring the deformation that occurs when a controlled crack is introduced into the material, and stripping methods that evaluate deformation while stepwise removal of the material layer to determine the residual stresses within the films and coatings [28,29,30]. Lastly, semi-destructive techniques combine the above two, including the hole-drilling; a well-established technique for residual stress measurement at different depths by drilling a hole within coating thickness [31,32,33,34] and the FIB-DIC ring-core milling is a high-resolution method for residual stress measurement at microscale by milling micro pillars or slots within the coating thickness [7,35,36,37,38,39,40,41].

**Figure 1:**
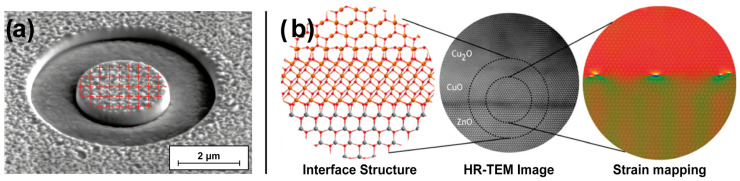
(**a**) Illustration of the FIB-DIC residual stress analysis method, showing a typical displacement tracking grid used for incremental strain measurement. (**b**) Application of the TEM GPA method to a Cu_2_O/ZnO interface (reproduced from [50]), showcasing atomic structure, HR-TEM imaging, and strain mapping to visualize strain-induced CuO formation at the interface (reproduced and adapted from [51]).

This study employs a multiscale approach to residual stress characterization in multilayer thin coatings by integrating HRSTEM-GPA (see Figure 1b) and FIB-DIC technique (see Figure 1a) for comparison due to their complementary high-resolution capabilities in nanoscale and microscale level. HRSTEM-GPA enables atomic-scale strain mapping to capture localized stress variations at interfaces, while FIB-DIC facilitates microscale stress analysis to assess the overall stress distribution across the coating thickness.

The HRSTEM-GPA provides quantitative information on elastic strain present in materials at the sub-nanometric scale, making it ideal for investigating local lattice distortions and interface-related stresses in multilayer coatings. Two types of image processing methods can be employed to extract deformation maps from lattice spacing variations across HRTEM images: geometric phase analysis (GPA), which relies on Fourier space information [42,43] and real-space peak-finding methods [44] that benchmark the position of intensity maxima in the area under analysis against the intensity distribution in a non-distorted lattice region. Both approaches yield similar results, however, real space algorithms typically require human intervention when dealing with the highly distorted dislocation cores, while GPA is not only straightforwardly employed but also generates deformation maps in agreement with the linear elastic theory of dislocations [45,46].

The Fourier Transform (FT) of a perfect lattice image gives rise to sharply peaked frequencies (Bragg spots). At the same time, local variations in interplanar distance produce diffuse intensity centered around the mean Bragg spots. GPA is based on filtering with numerical aperture-specific Bragg spots and corresponding diffuse intensity. The Fourier coefficients *H_g_* are allowed to become a function of position r in the image (*H_g_*(r)), and the Δg deviations can consequently be equated to an additional phase term *P*(*r*) in the Fourier coefficient mapped in real space. The fundamental assumption of the methodology lies in assuming that each set of real-space lattice fringes can be described by the mean frequency g with the local displacement of the sinusoidal function dictated by the phase. The vectorial displacement field can then be directly inferred from. Thus, phase maps of two non-colinear g can be used to generate strain maps by differentiation. The *P*(*r*) phase is called geometric as it pertains to changes in the position of lattice fringes in an image rather than to the phase of periodicities in the electron wavefunction emerging from the crystal.

In general, the reliability of strain mapping relies on the constant spatial relationship between the intensity maxima in the image and the relative positions of the atomic columns in the specimen, which is the hallmark of high-angle annular dark field images in high-resolution scanning transmission electron microscopy (HRSTEM). Nevertheless, the method is relative and does not allow us to determine the absolute distortion field present in the sample. In addition, it can be affected by thin foil relaxation, local crystal tilts, and thickness and/or composition variations.

The Focused Ion Beam—Digital Image Correlation (FIB-DIC) ring-core milling technique enables micro-scale residual stress evaluation, capturing stress distribution over a larger spatial range, typically from sub-micron to micron-scale. This allows for a broader assessment of stress relaxation mechanisms within the coating structure [47]. The technique combines incremental FIB ring-core milling, high-resolution scanning electron microscopy (HRSEM) imaging after each milling step, and surface displacement calculation using MATLAB-based DIC analysis (see Figure 1a) [48,49].

FIB micro-milling induces controlled strain relaxation at the stressed coating surfaces. Then the relaxation strain is measured at the coating’s surface of HRSEM micrographs using a DIC MATLAB-based routine, and the residual stress profile throughout the coating thickness is then calculated from a developed MATLAB-based routine (considering the number of coating layers, layer thicknesses, pillar diameter, relaxation strain, elastic modulus and poison’s ratio of both substrate and coating layers, respectively) [52]. The FIB-DIC method has been widely used and proved its accuracy for semi-destructive residual stress measurement of thin films [35,37,47,53,54].

In this article, the heterogeneous AlGaN and GaN multilayer coatings were deposited on a sapphire substrate using the metalorganic vapor phase epitaxy (MOVPE) method. These coatings exhibit excellent optical and structural properties that make them good candidates for optical applications such as optical sensors [55]. Some previous studies state that AlGaN/AlN/GaN heterogeneous structures grown on epitaxial AlN/Sapphire substrate show high electron Hall mobility of 2500 cm^2^/Vs that corresponds to efficient and fast charge transfer, essential for applications like transport conductive oxides, photovoltaic cells, and electronic devices [56]. The AlN/GaN/sapphire heterostructure shows a temperature coefficient of frequency (TCF) value of −34.6 ppm/°C, demonstrating the potential of the waveguiding layer acoustic waves (WLAW) that makes them suitable for high-temperature package less acoustic wave devices [57]. These high-quality, low-sheet-resistive layers are of key importance to avoid current crowding in quaternary AlGaN/GaN multiple-quantum-well deep-ultraviolet light-emitting diodes over sapphire substrates [58].

This study investigates residual stress distribution across multilayer coatings using a comparative assessment of two advanced characterization techniques: HRSTEM-GPA and FIB-DIC, which offer distinct spatial resolutions ranging from the sub-nanometer to the sub-micron scale. By critically evaluating their accuracy, efficiency, and compatibility, aims to establish a reliable framework for residual stress quantification in nanoscale multilayers. Furthermore, the integration of these techniques provides comprehensive insights into interfacial stress states, contributing to a deeper understanding of stress evolution mechanisms in complex coating architectures.

## 2. Results

### 2.1. X-Ray Diffraction

**Figure 2:**
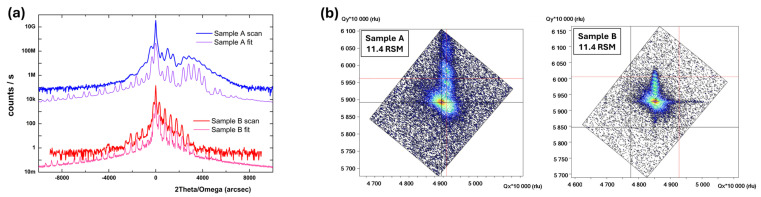
(**a**) HRXRD 2theta-omega 00.2 scans and simulations of the sample A—Al_0.48_Ga_0.52_N/GaN (in blue) and sample B—Al_0.22_Ga_0.78_N/GaN (in red) superlattices, (**b**) together with their 11.4 RSMs.

The X-ray diffraction 2θ-ω scans show the epitaxial growth of both sets of multilayer coatings on sapphire substrate, which makes them suitable for the HRSTEM-GPA analysis for the evaluation of the localized strain at the sub-nano level. The reciprocal space maps (RSMs) obtained from XRD as shown in Figure 2, confirm the epitaxial growth and progressive improvement in crystal quality of the AlGaN and GaN layers with increasing distance of the respective layers from the substrate. This enhancement is primarily attributed to a reduction in dislocation density and a decrease in mosaicity, as evidenced by the narrowing of the (11.4) reciprocal lattice points. The observed decrease in peak broadening suggests improved lattice coherence and strain relaxation, indicating a more defect-free and well-ordered crystal structure in the upper layers. The detailed sublayers geometrical growth and coating thicknesses of each layer are presented in Table 1.

The HRXRD 2θ-ω (00.2) scans for Sample A (Al_0.48_Ga_0.52_N/GaN, blue) and Sample B (Al_0.22_Ga_0.78_N/GaN, red) reveal distinct diffraction peaks corresponding to the periodic multilayer structures. The presence of well-defined superlattice satellite peaks in both samples indicates high-quality periodic stacking of AlGaN and GaN layers with good interface sharpness and structural coherence.

### 2.2. GPA Method

**Figure 3:**
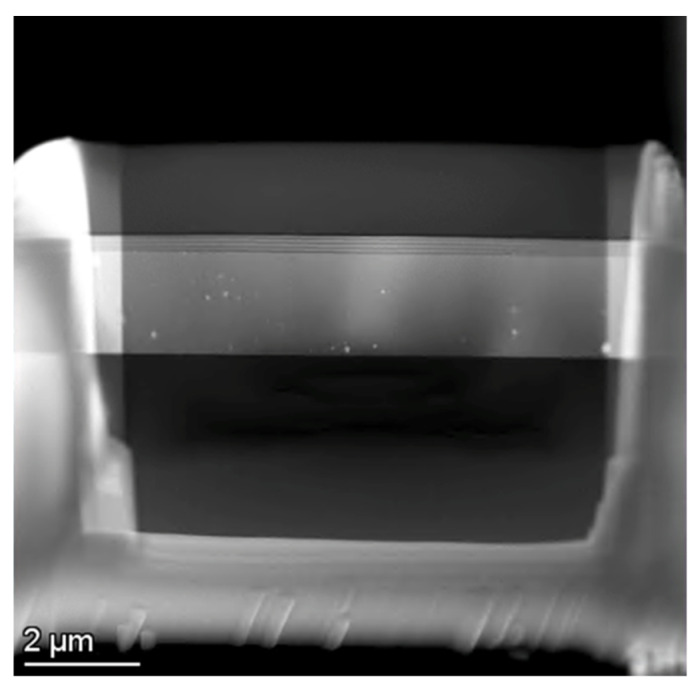
STEM lamella extracted from the Al_0.22_Ga_0.78_N/GaN sample.

The GPA can only be used reliably for localized strain mapping. Annealing was performed for 2 h at 600 °C on a piece of the CTN CT 4 layers sample to characterize the stress at the interfaces. However, the stress level was still at levels high enough to prevent an effective GPA. It was then decided to produce films with epitaxial interfaces. Then multilayers consisting of Al_0.48_Ga_0.52_N/GaN (A) and Al_0.22_Ga_0.78_N/GaN (B) were produced, sectioned by focused ion-beam for STEM and analyzed by GPA. The analysis was carried out in 5 regions of sample A. The GPA results indicate that the thin film exhibits residual compressive stress both in-plane and perpendicular to the substrate. The STEM lamella of Al_0.22_Ga_0.78_N/GaN was prepared by FIB as shown in Figure 3.

**Figure 4:**
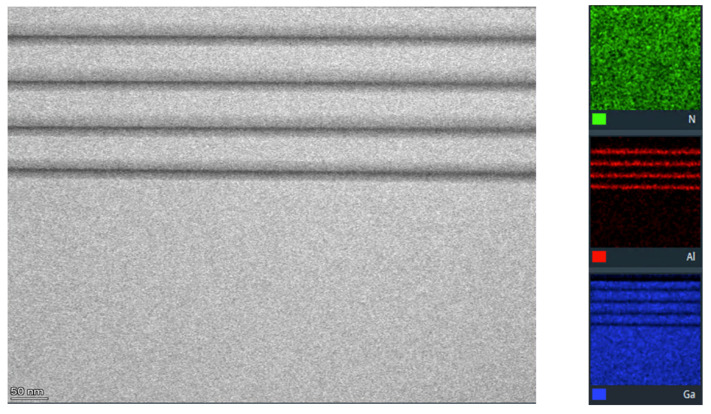
The SEM-EDS mapping for the evaluation of microstructure and elemental composition.

The Scanning Electron Microscopy with Energy Dispersive X-ray Spectroscopy (SEM-EDS) analysis was performed in order to determine the layered microstructure and identify the elemental composition of the layer’s material to assess its integrity and uniformity as shown in Figure 4. The SEM-EDS analysis confirms the expected elemental composition of the multilayer coating, and the color-coded maps show the distribution of the key elements (Al, Ga, and N) in the coating.

**Figure 5:**
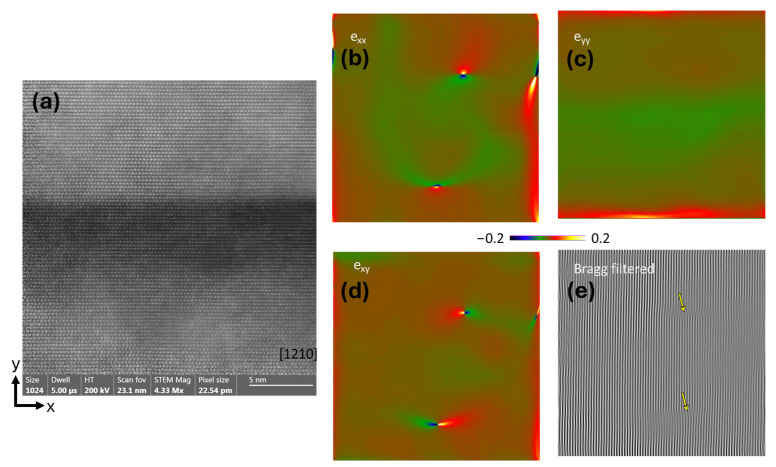
GPA performed in sample A—Al_0.48_Ga_0.52_N/GaN. (**a**) Lattice image of multilayer films. (**b**) e_xx_ image, (**c**) e_yy_ image, (**d**) e_xy_ image, where the color scale indicates the stress level. (**e**) Bragg’s filtered image of the same region where the yellow arrows point to the dislocation position.

**Figure 6:**
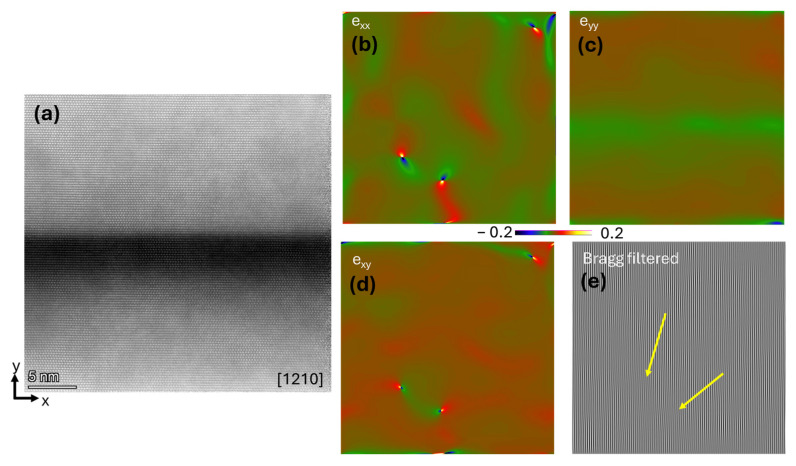
GPA performed in sample B—Al_0.22_Ga_0.78_N/GaN. (**a**) Lattice image of multilayer films. (**b**) e_xx_ image, (**c**) e_yy_ image, (**d**) e_xy_ image, where the color scale indicates the stress level. (**e**) Bragg’s filtered image of the same region where the yellow arrows point to the dislocation position.

Figure 5 and Figure 6 present illustrative examples of the GPA measurements performed on the Al_0.48_Ga_0.52_N/GaN (A) and Al_0.22_Ga_0.78_N/GaN (B), respectively. The colored images show the long-range stress fields emanating from misfit interfacial dislocations (b–d). Bragg-filtered images revealing the position of the edge dislocations are also presented (e).

The TEM-GPA method successfully maps the localized strain states associated with misfit dislocations in the two sets of multilayer coatings with different layer geometries (Sample A and B). The average stresses extracted from 5 different regions in each sample yielded the average stress state. These results are statistically limited due to the scale of lattice images. Still, they allow interpretation of the contribution of the localized interfacial strain to the overall residual stress of the coatings, as shown in Equation (1) for sample A and Equation (2) for sample B.

(1) $$\left[\begin{array}{cc} e_{xx} & e_{xy}\\ e_{xy} & e_{yy} \end{array}\right]=\left[\begin{array}{cc} -0.00339 & 0.00133\\ 0.00133 & -0.00743 \end{array}\right]$$

(2) $$\left[\begin{array}{cc} e_{xx} & e_{xy}\\ e_{xy} & e_{yy} \end{array}\right]=\left[\begin{array}{cc} -0.00454 & -0.00114\\ -0.00114 & -0.00746 \end{array}\right]$$

### 2.3. FIB-DIC Method

In the FIB-DIC ring-core residual stress measurement method, relaxation strains are extracted through MATLAB-based DIC analysis of HRSEM images acquired after each milling step. These relaxation strain data are then utilized in a MATLAB-based routine to compute the residual strain profiles. In previous studies, FIB-DIC method has been successfully applied to the vast variety of thin films and coatings for the evaluation of residual stresses across the coating thickness such as TiCuAg composite coatings [40], ZrCu metallic glass thin films [41], Ti-based multilayer coatings [9,59], and Cr-based multilayer coatings [52,60,61]. 

**Figure 7:**
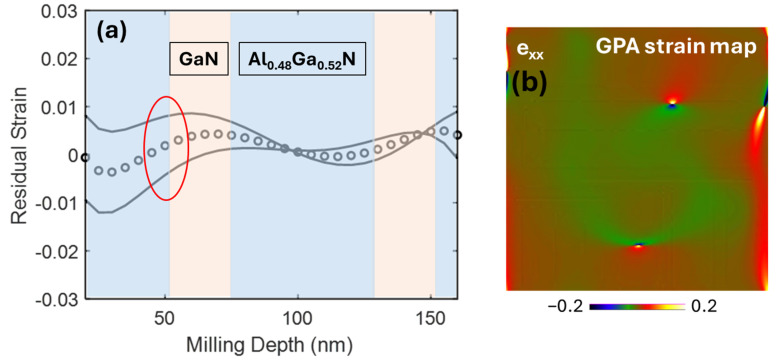
(**a**) FIB-DIC residual strain value 8.45 × 10^−4^ (±6.71 × 10^−3^/±5.05 × 10^−3^) (The GPA inspected region is highlighted in red circle), (**b**) TEM-GPA strain value (−3.39 × 10^−3^).

A comparison between the residual strain calculated by the FIB-DIC and HRSTEM-GPA method is presented in Figure 7. The sampling region of interest for the case of the TEM-GPA method is highlighted in red circles in residual strain profiles. The residual strain calculated by the FIB-DIC method in the GPA-inspected region is 8.45 × 10^−4^ (+6.71 × 10^−3^/−5.05 × 10^−3^), which is slightly different from −3.39 × 10^−3^ evaluated by the GPA method. This discrepancy may be attributed to the presence of dislocations in the GPA map.

**Figure 8:**
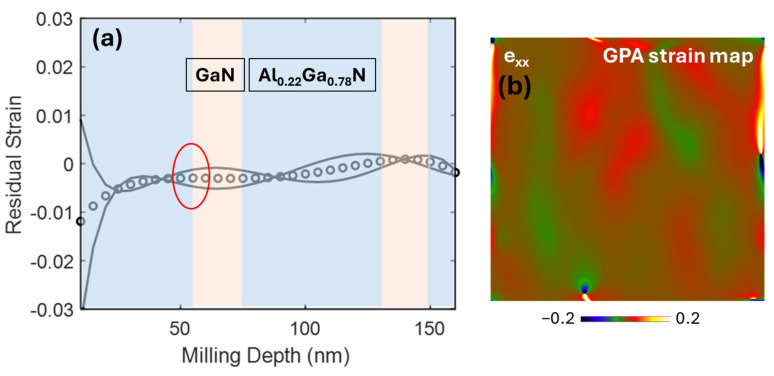
(**a**) FIB-DIC strain value −3.2 × 10^−3^ (±1.26 × 10^−3^/±4.62 × 10^−3^) (The GPA inspected region is highlighted in red circle), (**b**) TEM-GPA strain value (−4.55 × 10^−3^).

In the case of Sample B, there is an auspicious match between the residual strain values obtained by both techniques, FIB-DIC and TEM-GPA, respectively. The residual strain curves are presented in Figure 8, which shows positive strain values that correspond to the presence of compressive residual stresses within the coating thickness. The red encircled areas on all the strain profiles are the interfacial sampling region for the GPA method. The residual strain values, −3.2 × 10^−3^ (±1.26 × 10^−3^/±4.62 × 10^−3^), calculated by FIB-DIC and TEM-GPA methods show a strong validation and profound accuracy between both high-spatial resolution techniques.

## 3. Discussion

The structural properties of MOVPE-grown AlGaN/GaN multilayer coatings have been investigated using high-resolution x-ray diffraction (HRXRD) and reciprocal space maps (RSMs). The HRXRD 2θ-ω (00.2) scans, supported by simulations, confirm the epitaxial quality, compositional accuracy, and structural integrity of the AlGaN/GaN superlattices. The observed diffraction scan shows the (00.2) Bragg peak positions are in good agreement with the simulated spectra that demonstrates coherent layer growth, well-defined interfaces, and expected compositions. The two sets of coatings, 4-fold alternative sublayer geometry (AlGaN/GaN), different sublayer thickness with 1.7 µm GaN buffer layer, were deposited on the sapphire substrate by MOVPE method. The sublayer thicknesses and total thickness were measured by FIB cross-section; sample A and sample B have a total thickness of 296 nm and 276 nm, respectively. The residual strain values at the interfacial regions between the first layers within the heterogeneous multilayer coatings are calculated by using two advanced techniques with different spatial resolutions (from sub-micron to sub-nano scale), HRSTEM-GPA and FIB-DIC, respectively.

The epitaxial growth of these coatings is confirmed by HRXRD and RSMs techniques, which makes them suitable for the TEM-GPA analysis. HRXRD scans with the reflection of 00.2 were performed, and asymmetrical 11.4 RSM maps were obtained to determine the thickness, composition, relaxation, and crystal quality of the subsequently grown layers. The structural data were analyzed by means of curves fitting to the 2theta/omega scan of 00.2 reflections preceded by an analysis of reciprocal space maps around the 11.4 lattice point, which confirms its epitaxial growth [62,63].

### 3.1. Comparison Between These Two Techniques

In the case of sample B: Al_0.22_Ga_0.78_N/GaN coatings, similar residual strain values are obtained from both techniques (FIB-DIC and TEM-GPA; −3.2 × 10^−3^ and −4.55× 10^−3^), negative residual strain values corresponding to the presence of tensile stresses across the coating’s interfacial structure. Both high-spatial resolution techniques provide strong validation and accurate results for the same region of interest across the thicknesses. The residual strain values obtained from FIB-DIC (−3.2 × 10^−3^) and TEM-GPA (−4.55 × 10^−3^) reveal a consistent trend in the overall stress state of the multilayer coatings, though slight quantitative differences exist due to the inherent characteristics of each technique. HRSTEM-GPA resolves atomic-scale lattice distortions, making it more sensitive to localized strain concentrations at interfaces, which may result in higher strain values [64].

Despite their effectiveness in characterizing residual stresses at different length scales, HRSTEM-GPA and FIB-DIC have inherent limitations primarily related to sample preparation artifacts, measurement uncertainties, and spatial resolution constraints. HRSTEM-GPA provides atomic-resolution strain mapping, making it highly suitable for analyzing interface-related stresses in multilayer coatings. However, the technique is sensitive to FIB-induced damage during TEM lamella preparation, where ion milling can introduce implantation defects, amorphization, and local stress relaxation, potentially leading to underestimated or altered strain values [65]. Additionally, strain analysis in thin TEM specimens (<100 nm) may not fully represent the bulk stress state, as the thinning process can cause stress relaxation, especially in highly strained multilayers [64]. Other sources of uncertainty include STEM imaging distortions, reference lattice selection biases, and scan drift, all of which can affect strain quantification.

On the other hand, FIB-DIC enables microscale stress evaluation across larger regions, making it more applicable for coating-scale analysis. However, its accuracy is highly dependent on the quality of the speckle pattern, where poor contrast or non-uniform deformation markers can introduce errors in strain mapping [66]. Additionally, ion milling during the incremental material removal process may induce stress relaxation, leading to potential discrepancies between measured and actual residual stress values. The spatial resolution of FIB-DIC (~sub-micron) is also lower compared to HRSTEM-GPA, making it less effective in resolving localized interface stresses in nanoscale multilayers [29]. Given these limitations, a combined approach integrating both techniques can provide a multiscale assessment of residual stress, ensuring a more comprehensive and reliable characterization of thin film and multilayer coating systems.

The residual strain values measured for Al_0.48_Ga_0.52_N/GaN coatings (Sample A) using TEM-GPA and FIB-DIC reveal significant differences, highlighting the effect of spatial resolution and measurement principles on stress evaluation. The negative residual strain (−3.39 × 10^−3^) obtained via TEM-GPA suggests the presence of tensile residual stresses within the multilayer structure. In contrast, FIB-DIC results (8.45 × 10^−4^) indicate an almost stress-free state within the analyzed volume. This discrepancy is primarily attributed to the length scale of the respective techniques and their sensitivity to localized lattice distortions versus bulk stress distribution: this gives for sample A still valid comparison. The observed variation in calculated residual strain values may be attributed to the presence of dislocations in the GPA maps and the differences in spatial resolution between the techniques. Beyond these inherent differences, a key factor influencing the results could be the annealing process required prior to applying the GPA method. Unlike GPA, the FIB-DIC technique does not necessitate annealing, which may lead to discrepancies in the measured residual stress state. Studies have shown that thermal pre-treatment can induce stress relaxation, significantly altering the residual stress profile in multilayer coatings by promoting dislocation movement, defect annihilation, and strain redistribution. Additionally, differences in resolution and material removal strategies between the two techniques may further contribute to variations in the observed strain values [37].

### 3.2. Effect of Dislocation

The presence of dislocations strongly influences the residual strains within the multilayer coatings. The dislocations can act as stress-relief mechanisms in coatings as the lattice mismatch between the adjacent layers generates excessive strain dislocations from the interfaces to relieve this strain partially. The introduction of dislocations reduces the residual strain but can lead to localized distortions near the interfaces. This redistribution of strain depends on the density and type of dislocation (e.g., edge or screw dislocations). The network of misfit dislocation may develop at the interfaces within multilayers because of significant lattice mismatches. These dislocations accommodate the mismatch and minimize the buildup of elastic strain energy.

Such networks can stabilize the structure by reducing overall strain energy, but they may also introduce localized stress concentrations that could lead to failure under mechanical or thermal loading. The dislocations in multilayer thin coatings can either relieve or redistribute residual strain, depending on factors such as the lattice mismatch, layer thickness, temperature, and material properties. While they generally help with strain relaxation, they can also introduce localized stresses that may affect the mechanical integrity and durability of the coating system.

Environmental conditions, such as temperature fluctuations, humidity, vacuum pressure, and sample preparation conditions can significantly influence the accuracy and reliability of residual stress measurements obtained through HRSTEM-GPA and FIB-DIC. For both techniques, it is essential to control environmental parameters as much as possible to avoid introducing external errors that could compromise the precision of the strain measurements. These factors must be carefully considered during experimental design, data collection, and interpretation to ensure that the results reflect the true material behavior rather than artifacts induced by environmental influences.

Both are high-spatial resolution techniques ranging from sub-micro (FIB-DIC) to sub-nano scale (HRSTEM-GPA). The combination of these techniques provides us with more validated, trusted, and accurate results regarding the residual strain at the interfacial layers within the complex and heterogeneous coatings. The TEM-GPA method is restricted to the presence of polycrystallinity. Highly stressed multilayer coatings cannot be analyzed, while the FIB-DIC method can be applied to a wide range of materials irrespective of the crystal structures and stress state. The difference between the samples may not be significant, given the small sampling size associated with STEM analysis.

## 4. Materials and Methods

### 4.1. Production of Multilayer Coatings

The investigated structures have been epitaxially grown on single-side polished, two-inch diameter, 430 ± 15 µm thick sapphire substrates. The C-plane (0001)-oriented substrates with off-angle toward M-axis 0.2 ± 0.1° and toward A-axis 0 ± 0.1° have been chosen as a standard alternative substrate for epitaxial growth of nitrides. The quality of the substrates was defined by the supplier by the etch pits density (EPD) at the level lower than 5×10^8^/cm^2^, total thickness variation (TTV) ≤ 10 µm, and bowing < 10 µm. The growth of the periodical structures has been preceded by the metalorganic vapor phase epitaxy (MOVPE) method in a vertical flip-top closed coupled showerhead (FT 3 × 2 CCS) AIXTRON reactor designed for three 2’ diameter wafers. For the growth of nitrides, hydrogen (H2) was used as a carrier gas, and ammonia (NH3), trimethylgallium (TMGa), and trimethyl aluminum (TMAl) as precursors. Standard of 1.7 µm thick undoped GaN buffer layer and subsequent undoped Al_048_Ga_0.52_N/GaN (Sample A) or Al_0.22_Ga_0.78_N/GaN (Sample B) periodical structures have been grown at 50 mbar in the reactor and at 1060 and 1080 °C for the buffer and superlattices respectively. In situ, reflectance monitoring was performed using a Laytec EpiTT system operating with a 633 nm laser. Emissivity-corrected pyrometry (true temperature) was used to determine the sample surface temperature during growth. The schematic illustrations of the investigated multilayer coating structure geometry with the thickness measurement of each layer are presented in Table 1. The multilayer coatings exhibit four alternative sublayers of AlGaN and GaN with a total thickness of 296 nm and 276 nm for samples A and B, respectively. In both cases, a 1.7 µm thick buffer layer of GaN was used for better adhesion and growth of the coatings.

### 4.2. XRD Analysis

A high-resolution X-ray diffraction technique (HRXRD) was used to determine the structural data of each investigated sample. HRXRD scans of 00.2 reflections were performed, and asymmetrical 11.4 reciprocal space maps (RSMs) were obtained to determine the thickness, composition, relaxation, and crystal quality of the subsequently grown layers. All structural data have been determined by means of curves fitting to the 2theta/omega scan of 00.2 reflections preceded by an analysis of reciprocal space maps around the 11.4 lattice point. The Empyrean X-ray diffractometer equipped with Cu kα1 = 1.540597 Å wavelength source and supported by a Pixcel3D detector with a hybrid monochromator in incidence beam mode has been used for that investigation. The quality of the epitaxial samples, including interface sharpness and uniformity, was verified by transmission electron microscopy (TEM) imaging.

### 4.3. GPA Suitability

GPA can be successfully employed to map elastic strain provided the sample is not extremely deformed since the atomic columns need to be clearly discerned, and an unstrained region with the same crystallographic orientation is present in every field of view to be used as reference. The localized strain associated with coherent interfaces or semi-coherent interfaces with misfit dislocations is particularly suited for GPA. However, these extended defects must be observed in edge-on and end-on orientations, respectively. The AlGaN/GaN multilayer coatings produced by MOVPE exhibited localized strain at the interfaces and relatively defect-free regions in the layers, rendering them suitable for GPA. In contrast, the generalized high level of residual strain and small crystallite sizes in the samples prepared by sputtering precluded the use of GPA.

### 4.4. TEM Lamella Preparation

The lamellas of the multilayer samples produced by sputtering and pulsed laser deposition were precisely sectioned with a gallium-focused ion beam using an FEI Helios G4 Dual-Beam instrument (ThermoFisher Scientific, Hillsboro, Oregon, USA) at 30 kV and subsequently polished at 5 and 2 kV to remove amorphous layers.

### 4.5. High-Resolution—Scanning Transmission Electron Microscopy (HR-STEM) and Geometric Phase Analysis (GPA)

A DCOR Cs probe-corrected FEI Titan G2 60-300 instrument, with 0.08 nm nominal spatial resolution when operated at 200 kV, was used for the HRSTEM work. A high-angle annular dark field obtained with a convergence angle of 22 mrad was employed for image acquisition. GPA evaluated the distribution of misfit strain at the interfaces between the layers produced by PLD using the FRWR tools plugin (Frithjof (Frits) Radelaar, TU Delft, Delft, The Netherlands) for digital micrographs. This method was used to investigate type-III residual stresses (σRS, III) existing over atomic dimensions.

The key steps of GPA involved:

a Selection of two non-colinear reciprocal vectors g_1_ and g_2_ from the power spectrum.
b Selection of the size of the Gaussian mask (resolution).
c Selection of a reference image from which the positions of the mean g vectors were established and around which the masks were centered.

The two-phase maps were used to calculate the local displacement and map the strain components and rotation.

### 4.6. Focused Ion Beam—Digital Image Correlation (FIB-DIC) Method

The residual stress measurements were carried out by the FIB-DIC micro-ring core method on a FEI Helios Nanolab 600 dual beam Focused Ion Beam Scanning Electron Microscope (FIB/SEM, Thermo Fisher Scientific, Waltham, MA, USA), using a specifically developed automated procedure [35]. The milling was performed using an annular trench with an inner diameter of 2 µm while employing a current of 28 pA at the acceleration voltage of 30 kV. Ten high-resolution secondary electron images were acquired before and after each milling step using an integral of 150 images at a dwell time of 50 ns. The automatic procedure continuously monitored and corrected electron and ion beam drift while maintaining the same contrast of the reference image. The milling was performed until the h/D ratio of 0.2 was achieved, where h and D represent the milling depth and the pillar diameter, respectively. The h/D ratio of 0.2 ensures optimal strain relief, as demonstrated in several recent publications [7,54,67]. After the milling cycle, all the HRSEM images were processed with a customized MATLAB v2.1.0.0-based DIC code [68] to calculate the relaxation strain over the pillar surface. The residual stress profiles in the multilayer coatings were computed using a developed MATLAB-based routine that considered relaxation strain calculated by DIC as input and elastic modulus (E) and Poisson’s ratio (v) for both coatings and substrate material.

## 5. Conclusions

The residual stress distribution in AlGaN/GaN multilayer coatings was evaluated using two high-resolution techniques, HRSTEM-GPA and FIB-DIC, providing valuable insights at different spatial scales. Both methods consistently captured the overall trends in residual strain, revealing the epitaxial nature of the coatings. However, HRSTEM-GPA, with its sub-nanometer resolution, detected more localized lattice distortions at the interfaces, reflecting the finer details of stress accumulation, particularly at the atomic level. In contrast, FIB-DIC, with a sub-micron resolution, provided a more averaged strain response over a larger volume, which may have contributed to a lower strain measurement compared to HRSTEM-GPA.

A critical comparison was made between the residual strain measurements obtained using HRSTEM-GPA and FIB-DIC for the AlGaN/GaN multilayer coatings. Both techniques produced consistent results, indicating negative residual strain (−4.5 × 10^−3^), which corresponds to the presence of tensile residual stresses within the film for Sample B. Notably, both methods revealed the absence of dislocations in the GPA maps, supporting the uniformity of the strain distribution. The area of interest (AOI) was specifically selected at the interfaces of the first two layers, highlighted in red on the corresponding strain curves, allowing for a focused evaluation of the interfacial stress states. The consistency between the two techniques emphasizes their reliability in capturing the tensile stress profile, though differences in resolution and sampling volume may have influenced the local strain measurements in finer detail. The influence of dislocation is quite evident in the case of sample B exhibiting the two dislocations on the GPA map; for that reason, the residual strain calculated by the HRSTEM-GPA method is −3.39 × 10^−3^ while the FIB-DIC method shows a strain value of 8.45 × 10^−4^. The reason for the change in results is the presence of dislocation on the selected AOI, which can be avoided for similar results in sample B with no dislocation.

HRSTEM-GPA is particularly advantageous for industries where atomic-level precision is paramount, such as in the semiconductor, optoelectronics, and microelectronics sectors. This technique’s ability to resolve strain at the atomic scale makes it ideal for assessing interface-related residual stresses, which are often crucial in the performance of heterostructures, and multi-layered materials used in high-performance transistors, laser diodes, and LEDs. For example, in the fabrication of AlGaN/GaN high electron mobility transistors (HEMTs), HRSTEM-GPA can be used to precisely measure strain at the interface between different material layers, providing critical insights into potential dislocation formation, strain relaxation, and device performance. Furthermore, this technique is valuable for quality control during device fabrication where the epitaxial growth quality of the layers directly impacts device performance. On the other hand, FIB-DIC is particularly suited for applications where larger sampling areas and microscale precision are needed. This method is often employed in industries such as automotive, aerospace, and coating technologies for high-performance materials used in engines, turbine blades, and protective coatings. The FIB-DIC technique offers a comprehensive assessment of the overall coating stress state, which is critical in ensuring the durability and integrity of parts subjected to cyclic loading and thermal fatigue. The microscale resolution allows for the analysis of larger sample volumes, offering a better understanding of the stress distribution across the thickness of coatings.

Both HRSTEM-GPA and FIB-DIC are valuable and complementary techniques for residual stress characterization. The integration of these high-resolution methods allows for precise measurement of residual stresses at the sub-micron to sub-nano scale, providing reliable and validated stress profiles. This comparative study offers significant insights that can aid the scientific community in selecting optimal coating synthesis methods and deposition parameters, ultimately enabling the production of coatings with enhanced structural durability tailored to specific applications. The outcomes of this work lay the foundation for future investigations into the residual stress behavior of nanostructured multilayer coatings subjected to various annealing processes. Additionally, the methodologies and findings from this study can be applied across a wide range of industrial applications, providing valuable guidance for improving the performance and longevity of coatings in critical environments.

## Figures and Tables

**Table 1:** The structural data of the investigated samples A and B.

		Sample A			Sample B		
		Material	Thickness	Relaxation	Material	Thickness	Relaxation
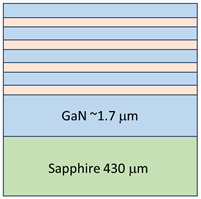	Super-lattice	GaN	52.0 nm		GaN	54.5 nm	
		Al_0.48_Ga_0.52_N	22.0 nm	0% to GaN buffer	Al_0.22_Ga_0.78_N	14.5 nm	0% to GaN buffer
	Buffer	GaN	1.7 µm	100% to sapphire	GaN	1.7 µm	100% to sapphire

## Data Availability

Data will be made available on request.

## Abbreviations

The following abbreviations are used in this manuscript:

FIB	Focused Ion Beam
DIC	Digital Image Correlation
HRSTEM	High-Resolution Scanning Transmission Electron Microscopy
GPA	Graphical Phase Analysis
AOI	Area of Interest
